# Mediators of the Association Between Severity of Noncommunicable Diseases and Subjective Health‐Related Quality of Life

**DOI:** 10.1002/npr2.70040

**Published:** 2025-07-23

**Authors:** Kazuyuki Nakagome, Michiyo Azechi, Teruo Noguchi, Chisato Izumi, Tsutomu Tomita, Fumihiko Yasuno, Reiko Saika, Yuji Takahashi, Hiroe Kikuchi, Maiko Fujimori, Yosuke Uchitomi, Yoshie Omachi, Yasunori Morio, Ryo Kanzaka, Mari Oba, Kotaro Hattori

**Affiliations:** ^1^ National Center of Neurology and Psychiatry Tokyo Japan; ^2^ Department of Psychiatry National Cerebral and Cardiovascular Center Osaka Japan; ^3^ Department of Cardiovascular Medicine National Cerebral and Cardiovascular Center Osaka Japan; ^4^ Department of Heart Failure and Transplantation National Cerebral and Cardiovascular Center Osaka Japan; ^5^ NCVC Biobank National Cerebral and Cardiovascular Center Osaka Japan; ^6^ Department of Psychiatry National Center for Geriatrics and Gerontology Morioka Japan; ^7^ Department of Neurology National Center of Neurology and Psychiatry Tokyo Japan; ^8^ Department of Psychosomatic Medicine National Center for Global Health and Medicine Tokyo Japan; ^9^ Division of Survivorship Research National Cancer Center Institute for Cancer Control Tokyo Japan; ^10^ Department of Cancer Survivorship and Digital Medicine The Jikei University School of Medicine Tokyo Japan; ^11^ Department of Forensic Psychiatry National Center of Neurology and Psychiatry Tokyo Japan; ^12^ Department of Clinical Research Support, Clinical Research & Education Promotion Division National Center of Neurology and Psychiatry Tokyo Japan; ^13^ Department of Bioresources, Medical Genome Center National Center of Neurology and Psychiatry Tokyo Japan

**Keywords:** cytokines, health‐related quality of life, heart failure, inflammation, mental health

## Abstract

**Aim:**

Noncommunicable diseases (NCDs) represent one of the greatest global burdens of disease and disability, and there is evidence that mental disorders associated with NCDs may reduce quality of life (QOL). We investigated the factors mediating the association between the severity of NCDs and subjective health‐related QOL in 173 patients with NCDs.

**Methods:**

We hypothesized that mental health indicators and inflammatory cytokines mediate the association between physical disease severity and subjective health‐related QOL. We conducted a structural equation model analysis and selected variables representing mental health and inflammatory cytokines using a multivariable regression analysis and factor analysis.

**Results:**

The structural equation model showed that mental health indicators such as anxiety and positive emotions are potential mediators, and that proinflammatory cytokines such as interleukin‐6 (IL6) and tumor necrosis factor‐α (TNF‐α) may reduce subjective health‐related QOL by increasing anxiety and suppressing positive emotions, without being particularly related to physical disease severity. The findings also suggest that anti‐inflammatory cytokines such as interleukin‐10 (IL10) and adiponectin (ADPN) are activated as physical disease severity increases, and likely protect against physical disease by enhancing positive emotions, potentially increasing subjective health‐related QOL and resilience.

**Conclusion:**

Mental health mediates the association between physical disease and subjective health‐related QOL, and between inflammatory cytokines and subjective health‐related QOL. Anti‐inflammatory cytokines are activated by physical disease severity and have a protective effect on mental health.

## Abstract 2

Mental health mediates the association between physical disease and subjective health‐related quality of life (QOL), and between inflammatory cytokines and subjective health‐related QOL. Anti‐inflammatory cytokines are activated by physical disease severity and have a protective effect on mental health.

## Graphical abstract

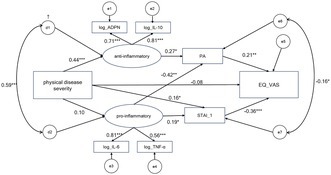

## 1 Introduction

Noncommunicable diseases (NCDs) represent one of the most important and neglected global disease burdens. According to the World Health Organization (WHO) report, NCDs are diseases caused by genetic, physiological, environmental, and behavioral factors that cannot be transmitted from person to person [1]. In 2019, the WHO recorded that 17 million people died from an NCD before the age of 70 years, accounting for 74% of all deaths worldwide [2]. Global trends driven by demographic changes and aging populations are expected to increase the number of patients with NCDs, as well as mental health and neurological conditions [1].

NCDs account for both “years of life lost” and “years lost due to disability” in the disability‐adjusted life years measure, and there is a major unmet need for improved quality of life (QOL) in NCD patients. Mental disorders associated with NCDs may reduce QOL in NCD patients.

Many studies have identified associations between mental and physical conditions, including myocardial infarction prognosis and the presence or absence of depression [3, 4, 5], the high rate of comorbid depression in diabetes [6, 7], and depression as a risk factor for dementia [8], but the relationship between physical diseases and psychological factors has not been fully elucidated. In recent years, it has been suggested that mental disorders may be caused not only by the central nervous system, but also by systemic factors such as inflammation, immunity, and glucose and lipid metabolism [9], and examining mental health in NCD patients may help to clarify the systemic factors that cause mental disorders and subjective distress. Therefore, the examination of bidirectional relationships between NCDs and mental disorders is an important issue that could inform efforts to extend healthy life expectancy and improve subjective QOL.

Chronic heart failure (CHF) can be considered to constitute not just a “defective pump” but a systemic disease [10]. Proinflammatory cytokines produced and released as a result of compensatory overactivity of the hormones and activation of the inflammatory system lead to a prolonged inflammatory state and reduced cardiac function [10]. There is evidence that elevated levels of various proinflammatory cytokines are associated with depressive symptoms [11]. Other NCDs are also associated with an activated inflammatory response and higher levels of inflammatory cytokines [12, 13, 14, 15] Across diagnoses, it is possible that mental health status in NCD patients may be mediated through inflammatory system activation. Physical comorbidities lead to poor treatment response in depression [12], suggesting that identification of mediating factors in mental distress, which can also lead to poor subjective QOL, could inform the development of treatments.

The present study was conducted as a Mental Health Registry study by the National Research Centers for Advanced and Specialized Medical Care in Japan. All the centers collected clinical information, including mental health data and blood samples in their respective areas of expertise: CHF, type 1 diabetes mellitus (T1DM), Alzheimer's disease (AD), mild cognitive impairment (MCI), Parkinson's disease (PD), and spinocerebellar degeneration (SCD). The clinical information was collected on a cloud database, and blood samples were processed and managed at each research center's biobank. The aim of this study was to identify pathways from NCDs to subjective QOL, a relationship that is possibly mediated by proinflammatory and anti‐inflammatory cytokines and different components of mental health. We hoped that the study would help to identify the physiological and pathological mechanisms underlying mental health and lead to the development of new mental and physical treatments.

## 2 Methods

### 2.1 Participants

**FIGURE 1:**
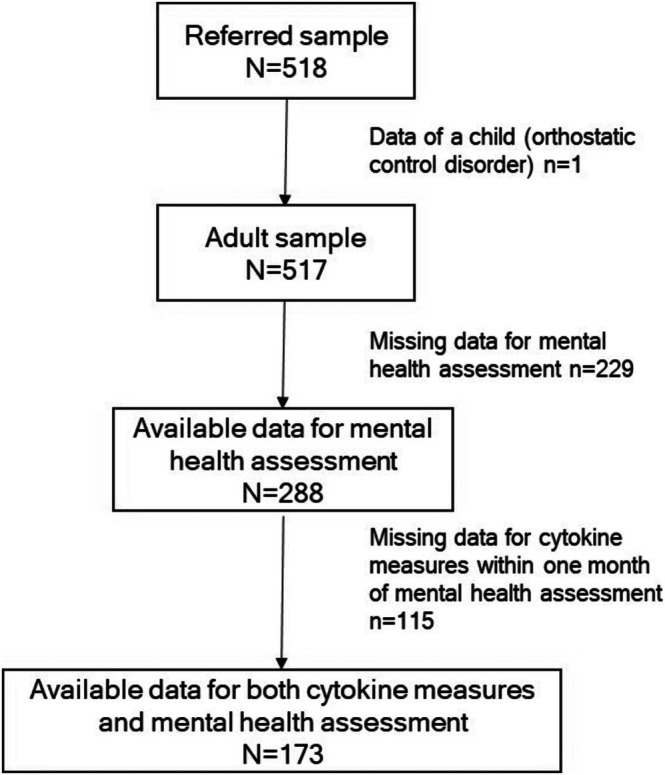
Flow diagram. Initially, 519 patients were enrolled in the study. As the number of orthostatic control disorders was only one and because of the age difference, the patient data from the NCCHD were excluded from the study. Next, patients from the NCC (*n* = 229) were excluded owing to different methods of mental health assessment in the NCC. Finally, data were excluded if the clinical assessment and blood sampling were not performed within 1 month (*n* = 115). NCC, National Cancer Center; NCCHD, National Center for Child Health and Development.

A total of 519 patients with NCDs were enrolled in the study between March 1, 2021, and March 31, 2023, and provided informed consent. Participants were either inpatients or outpatients at the six National Research Centers for Advanced and Specialized Medical Care in Japan: the National Cancer Center (NCC), National Cerebral and Cardiovascular Center (NCVC), National Center of Neurology and Psychiatry (NCNP), National Center for Global Health and Medicine (NCGM), National Center for Child Health and Development (NCCHD), and National Center for Geriatrics and Gerontology (NCGG). Patients with NCDs were those with head and neck cancer, esophageal cancer, and colorectal cancer, and elderly preoperative patients from the NCC; cardiovascular disease with heart failure from the NCVC; PD and SCD from the NCNP; T1DM from the NCGM; orthostatic dysregulation from the NCCHD; AD and MCI from the NCGG. However, patients from the NCC and the NCCHD were excluded from this study because of the different mental health assessment methods used in the NCC and the age difference of NCCHD participants. In addition, data were excluded if the clinical assessment and blood sampling were not performed within 1 month, which was the usual interval between each hospital visit (Figure 1).

The Ethics Committee of the NCNP approved the study protocol and experimental procedures (B2020‐110).

## 3 Assessments

Mental health, QOL representing self‐rated general health, and physical disease severity were assessed, and blood samples were taken within 1 month.

Physical disease severity was assessed by the treating physician using disease‐specific scales such as the New York Heart Association (NYHA) [13] functional classification for CHF, modified Rankin Scale (mRS) [14] for PD and SCD, Mini‐Mental State Examination (MMSE) [15] for AD and MCI, and hemoglobin A1C levels for T1DM, as shown in Table 1. These indicators were chosen because they are commonly used in routine clinical practice and do not impose an additional burden on patients or clinicians. Each assessment was translated into four levels of severity according to previous reports [13, 14, 15, 16].

**TABLE 1:** Physical diseases and their associated measurement scales and variables, translated into general severity levels.

Disease	Scales and variables	Score value	Severity
CHF	NYHA functional classification	I II III IV	1 (Normal) 2 (Mild) 3 (Moderate) 4 (Severe)
PD, SCD	mRS	0 1–2 3–4 5–6	1 (Normal) 2 (Mild) 3 (Moderate) 4 (Severe)
T1DM	HbA1c (%)	< 6.0 < 7.0 < 8.0 ≥ 8.0	1 (Normal) 2 (Mild) 3 (Moderate) 4 (Severe)
AD, MCI	MMSE	≥ 24 21–23 11–20 0–10	1 (Normal) 2 (Mild) 3 (Moderate) 4 (Severe)

Abbreviations: AD, Alzheimer's disease; CHF, chronic heart failure; HbA1c, hemoglobin A1C; MCI, mild cognitive impairment; MMSE, Mini‐Mental State Examination; mRS, modified Rankin Scale; NYHA, New York Heart Association; PD, Parkinson's disease; SCD, spinocerebellar degeneration; T1DM, type 1 diabetes mellitus.

Mental health was assessed using self‐reported assessments of affect, personality, and anxiety. The following self‐administered questionnaires were used: the Positive and Negative Affect Schedule (PANAS) [17] to measure positive and negative affect (NA), the Behavioral Inhibition and Activation Systems (BIS‐BAS) [18, 19] to measure motivational drives to activation and inhibition, and the State‐Trait Anxiety Inventory (STAI) [20, 21] Form X‐I and Form X‐II to measure state and trait anxiety. QOL was assessed using the EuroQol‐5 dimensions 5‐level (EQ‐5D‐5L) [22, 23] visual analog scale (VAS).

### 3.1 PANAS

The Japanese version of the PANAS [24] consists of 16 items that include adjectives describing positive and NA. The reliability and validity of the Japanese version of the PANAS have been demonstrated [24]. The eight positive affect (PA) items are excited, strong, enthusiastic, proud, alert, inspired, determined, and active. The eight NA items are distressed, upset, scared, irritable, ashamed, nervous, jittery, and afraid. PA and NA scores are calculated separately.

### 3.2 BIS‐BAS

The self‐report BIS‐BAS scales comprise a seven‐item BIS subscale and a 13‐item BAS factor comprising three subscales that assess motivational drives toward activation and inhibition [18, 19]. The BIS‐BAS is rated on a five‐point Likert scale from 1 = “does not describe me at all” to 5 = “describes me completely”; higher scores indicate greater BIS‐BAS sensitivity. The BIS subscale includes items related to responses to the anticipation of punishment. The BAS factor assesses how people respond to potentially rewarding events and consists of three subscales: Reward Responsiveness, Drive, and Fun Seeking.

### 3.3 STAI

The STAI consists of the STAI Form X‐I (STAI_1) and Form X‐II (STAI_2); the former assesses anxiety as a situational state and the latter assesses anxiety as a relatively stable personality trait [23]. STAI_1 and STAI_2 have been translated into Japanese and validated with 618 college students [24]. Each subscale consists of 20 items (including reverse items) that comprise short statements about an individual's subjective feelings. Respondents are asked to select one of four response options for each item: 1 = not at all/almost never, 2 = somewhat/sometimes, 3 = moderately so/often, and 4 = very much so/almost always. The minimum score for each scale is 20 and the maximum is 80; higher scores indicate greater anxiety.

### 3.4 EQ‐5D‐5L

The EQ‐5D‐5L consists of two parts. One part comprises EQ‐5D utility scores, which are a classification system of five dimensions (5D; mobility, self‐care, usual activities, pain/discomfort, and anxiety/depression) with five levels of response options (5 L; no problems, slight problems, moderate problems, severe problems, and unable to/extreme problems) per dimension [22, 23]. The other part comprises a VAS. In this study, we used the VAS (EQ_VAS), which records respondents' current self‐rated general health on a line ranging from 0 (the worst imaginable health) to 100 (the best imaginable health). We opted for VAS scores over EQ‐5D utility scores because we focused on the subjective distress, which is better reflected by the former scale.

### 3.5 Blood Samples

For plasma extraction, whole blood was drawn from each patient into ethylenediaminetetraacetic acid tubes at each institute. The blood samples were then centrifuged at 1710–3000 *g* for 5–15 min at 4°C or room temperature (centrifugation conditions varied at each site according to the rules of each research center's biobank). After centrifugation, 0.2–0.8 mL of plasma was aliquoted into 1–2 mL tubes and stored at −80°C in each institute's biobank, which varied from institute to institute.

Plasma concentrations of interleukin‐6 (IL‐6), tumor necrosis factor‐α (TNF‐α), interleukin‐10 (IL‐10), C‐reactive protein (CRP), interleukin‐1β (IL‐1β), adiponectin (ADPN), and leptin (LEP) were measured at Acel Inc. (Tokyo, Japan), using a Luminex Human Discovery Assay (R&D Systems Inc., Minneapolis, USA, #LXSAHM‐07) according to the manufacturer's instructions on a Bio‐Plex 200 system (Bio‐Rad Laboratories Inc., Tokyo, Japan) with low‐photomultiplier tube settings. Analyte concentrations were calculated using the Bio‐Plex Manager software (version 6.2.0.175, Bio‐Rad Laboratories Inc., Tokyo, Japan). Individual standard curves (straight lines) were generated based on the appropriate range of standards and fluorescence intensity, and after conversion to concentration, interplate correction was performed using the measured values of the pooled samples.

## 4 Statistical Analysis

The Kolmogorov–Smirnov test was used to assess the normal distribution of cytokine blood levels.

Structural equation modeling (SEM) with latent variables was used to examine the mediation effects of cytokines and mental health indicators on the association between physical disease severity and QOL. In the analysis, physical disease severity was the independent variable, cytokines and mental health indicators were the mediating variables, and QOL representing self‐rated general health was the dependent variable. In model testing, methods of assessing model fit were selected based on established criteria, including: (i) a nonsignificant chi‐square value, (ii) the comparative fit index (CFI), in which values > 0.95 are excellent and those > 0.90 indicate adequate fit [25], and (iii) the root mean square error of approximation (RMSEA), in which values ≤ 0.05 are considered good fit and those < 0.08 indicate adequate fit [26].

To explore which mental health indicators showed strong associations with QOL, multivariable regression analysis was performed with disease (CHF vs. others), age, sex, body mass index, PA, NA, BAS, BIS, STAI_1, STAI_2, and physical disease severity as independent variables and EQ_VAS score as the dependent variable, adjusting for possible confounders. In addition, a maximum likelihood factor analysis using Promax rotation was performed with cytokine variables to select those to be summarized in latent variables (labeled proinflammatory and anti‐inflammatory cytokines). The number of factors was determined using parallel analysis, comparing eigenvalues of the observed data with the 95th percentile of those derived from random datasets. Moreover, correlation analysis between QOL and other variables using Pearson's r was used to determine which variables were likely to be included in the SEM. IBM SPSS Statistics 30.0.0.0, Amos 29.0.0 (IBM Corp., Armonk, NY, USA), and SAS 9.4 (SAS Institute, Cary, NC, USA) were used for performing the data analyses.

## 5 Results

### 5.1 Patient Characteristics

The participants were 173 patients with NCDs, of whom 146 were CHF patients (ischemic heart disease: 59, others: 87), 14 had AD, four had MCI, three had T1DM, five had SCD, and one had PD (Table 2). A comparison of CHF patients with the rest of the group identified significant between‐group differences in age, sex, employment status, PA, ADPN, CRP, IL‐6, IL‐10, IL‐1β, LEP, and TNF‐α levels. Compared with patients with other conditions, those with CHF had lower PA and lower LEP levels, but higher levels of all other cytokines. Among patients with CHF, there were 51 patients in NYHA class I, 39 in class II, 14 in class III, and 42 in class IV. There was considerable overlap in complications between each group of diseases; 12 of the patients with CHF had complications of endocrine‐metabolic diseases, and 6 of these 12 patients had diabetes. In addition, 12 of the patients whose primary disease was not CHF had complications of cardiovascular disease.

**TABLE 2:** Comparison of demographics, clinical variables, and plasma cytokine levels between patients with CHF and patients without CHF.

Characteristic		Total population	CHF patients	Non‐CHF patients	*p*
No. of participants		173	146 (84.4%)	27 (15.6%)	
Main diagnosis			IHD: 59, others: 87	AD: 14, MCI: 4, T1DM: 3, SCD: 5, PD: 1	
Noncommunicable disease complications			EMD: 12^a^, MD: 2	CVD: 12, EMD: 7, MD: 1	
Age	Mean (SD)	58.9 (16.8)	56.8 (16.1)	70.1 (16.2)	< 0.001^b^
Sex	Male: *n* (%)	118 (68.6%)	108 (74.4%)	10 (37.9%)	
	Female: *n* (%)	54 (31.4%)	37 (25.5%)	17 (63.0%)	< 0.001^d^
BMI	Mean (SD)	22.5 (4.2)	22.7 (4.3)	21.8 (4.0)	0.351^b^
Employment status	Employed/at school: *n* (%)	80 (46.2%)	77 (52.7%)	4 (14.8%)	
	Not employed/at school: *n* (%)	93 (53.8%)	69 (47.3%)	23 (85.2%)	< 0.001^d^
Marital status	With partners: *n* (%)	128 (74.0)	112 (76.7%)	16 (59.3%)	
	Without partners: *n* (%)	45 (26.0%)	34 (23.3%)	11 (40.7)	0.058^d^
Physical disease severity	Mean (SD)	2.3 (1.2)	2.3 (1.2)	2.2 (0.9)	0.410^c^
PA	Mean (SD)	18.2 (7.7)	17.1 (7.4)	23.7 (7.4)	< 0.001^b^
NA	Mean (SD)	19.7 (8.7)	19.9 (8.8)	18.9 (8.2)	0.612^b^
BIS	Mean (SD)	18.4 (4.5)	18.3 (4.4)	18.8 (4.9)	0.590^b^
BAS	Mean (SD)	33.3 (8.7)	33.6 (8.8)	31.7 (7.7)	0.287^b^
STAI_1	Mean (SD)	43.2 (10.4)	43.7 (10.5)	40.5 (9.8)	0.145^b^
STAI_2	Mean (SD)	42.2 (10.1)	42.1 (9.6)	42.4 (12.5)	0.888^b^
EQ_VAS score	Mean (SD)	63.2 (22.8)	63 (23.5)	64.5 (19.1)	0.754^b^
ADPN (pg/mL)	Mean (SD)	640242.8 (36034.7)	643438.1 (36187.1)	622964.0 (30326.6)	0.006^b^
CRP (pg/mL)	Mean (SD)	63873.0 (6179.9)	64295.4 (5760.6)	61589.0 (7820.9)	0.036^b^
IL‐6 (pg/mL)	Mean (SD)	7.2 (5.0)	7.6 (5.2)	4.8 (3.0)	< 0.001^c^
IL‐10 (pg/mL)	Mean (SD)	4.4 (0.6)	4.4 (0.6)	4.0 (0.5)	0.001^b^
IL‐1β (pg/mL)	Mean (SD)	4.9 (1.5)	5.2 (1.5)	3.4 (0.8)	< 0.001^c^
LEP (pg/mL)	Mean (SD)	2240.1 (2574.8)	2071.8 (2454.7)	3150.6 (3038.7)	0.045^b^
TNF‐α (pg/mL)	Mean (SD)	9.3 (3.1)	9.7 (3.1)	6.8 (1.3)	< 0.001^c^

Abbreviations: AD, Alzheimer's disease; ADPN, adiponectin; BIS‐BAS, behavioral Inhibition and activation systems; BMI, body mass index; CHF, chronic heart failure; CRP, C‐reactive protein; CVD, cardiovascular disease; EMD, endocrine‐metabolic disease; EQ_VAS, EuroQol‐5 dimensions 5‐level visual analog scale; IHD, ischemic heart disease; IL, interleukin; LEP, leptin; MCI, mild cognitive impairment; MD, mental disorders; NA, negative affect; PA, positive affect; PD, Parkinson's disease; SD, standard deviation; STAI, State–Trait Anxiety Inventory; T1DM, type 1 diabetes mellitus; TNF‐α, tumor necrosis factor‐α.

^a^ Including six patients with diabetes mellitus.

^b^ Student's *t*‐test.

^c^ Welch's *t*‐test.

^d^ Chi‐square test.

### 5.2 Clinical Variables With a Strong Relationship to QOL

Multivariable regression analysis using EQ_VAS score as the dependent variable showed that PA was positively and STAI_1 negatively significantly associated with EQ_VAS, and physical disease severity showed a significant trend in association with EQ_VAS scores after adjustment for other variables (Table 3).

**TABLE 3:** Results of multivariable regression analysis using EQ_VAS scores as a dependent variable, and disease, age, sex, BMI, physical disease severity, and mental health indicators as independent variables.

Independent variables	Category	Ref	*β*	CI	*p*
Disease	CHF	Without CHF	3.17	[−6.7478, 13.0897]	0.531
Age			−0.11	[−0.3472, 0.1219]	0.347
Sex	Female	Male	1.81	[−5.4168, 9.0285]	0.624
BMI			−0.22	[−0.9872, 0.5472]	0.574
PA			0.68	[0.2324, 1.1263]	0.003**
NA			−0.25	[−0.7128, 0.2046]	0.278
BIS			−0.15	[−0.9917, 0.6922]	0.727
BAS			0.30	[−0.155, 0.7464]	0.199
STAI_1			−0.49	[−0.9208, −0.0567]	0.027*
STAI_2			−0.30	[−0.7549, 0.1604]	0.203
Physical disease severity			−2.41	[−5.263, 0.4375]	0.097

Abbreviations: BIS‐BAS, Behavioral Inhibition and Activation Systems; BMI, body mass index; CHF, chronic heart failure; CI, confidence interval; EQ_VAS, EuroQol‐5 dimensions 5‐level visual analog scale; NA, negative affect; PA, positive affect; STAI, State–Trait Anxiety Inventory.

* *p* < 0.05.

** *p* < 0.01.

### 5.3 Factor Analysis With Cytokine Data

The results of the Kolmogorov–Smirnov test were statistically significant, indicating that the cytokine data were not normally distributed. Therefore, a logarithmic transformation was performed for each variable.

A factor analysis with logarithmic cytokine variables identified three factors accounting for 70.7% of the variability among the variables (Table 4). Using a threshold loading value of 0.4 on the rotated loading, the first factor showed high factor loadings for two variables, log_IL‐6 and log_TNF‐α, labeled as the “proinflammatory” factor. The second factor showed high factor loadings for four variables, with the anti‐inflammatory cytokine IL_10 showing the highest value. Although proinflammatory cytokines such as log_CRP and log_IL‐1β were also included in this factor, it is noteworthy that log_ADPN, the other anti‐inflammatory cytokine [27], was included in the same factor as log_IL‐10. The third factor showed a high factor loading for log_LEP, which may be related to metabolic status.

**TABLE 4:** Results of a factor analysis with logarithmic cytokine variables.

	I	II	III
log_IL‐6	**1.08**.	−0.18	−0.01
log_TNF‐α	**0.42**	0.31	0.35
log_IL‐10	0.05	**0.70**	−0.20
log_CRP	−0.25	**0.59**	0.22
log_IL‐1β	0.20	**0.56**	0.07
log_ADPN	0.10	0.**53**	−0.27
log_LEP	0.10	0.05	0.**55**
Interfactor correlation	I	II	III
I	ー	0.51	−0.23
II		ー	−0.35
III			ー

*Note:* Bold text indicates relatively high factor loadings (> 0.4) for each factor.

Abbreviations: ADPN, adiponectin; CRP, C‐reactive protein; IL, interleukin; LEP, leptin; TNF‐α, tumor necrosis factor‐α.

### 5.4 Correlation of Clinical and Cytokine Variables

The multivariable regression analysis and the factor analysis indicated that PA, STAI_1, log_ADPN, and log_IL‐10 (anti‐inflammatory cytokines) and log_IL‐6 and log_TNF‐α (proinflammatory cytokines) were likely candidate variables for mediators between physical disease severity and EQ_VAS score.

**FIGURE 2:**
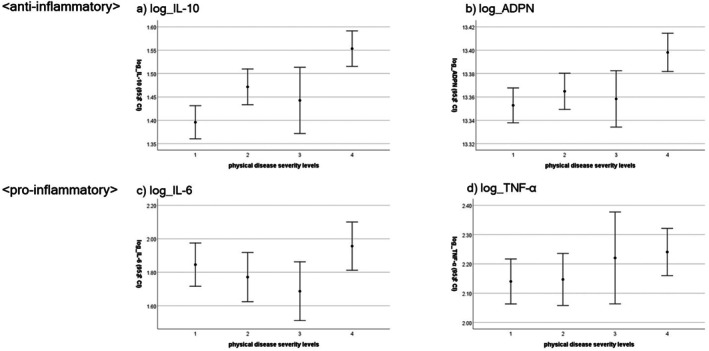
Proinflammatory and anti‐inflammatory cytokines and physical disease severity. Physical disease severity level 1: *N* = 60; 2: *N* = 45; 3: *N* = 25; 4: *N* = 43. Vertical lines attached to means represent 95% confidence intervals. Physical disease severity showed a significant correlation with the logarithmic transformed anti‐inflammatory cytokines (ADPN, IL‐10), but not with the proinflammatory cytokines (IL‐6, TNF‐α). ADPN, adiponectin; IL, interleukin; TNF‐α, tumor necrosis factor‐α.

Physical disease severity showed a significant correlation with the anti‐inflammatory cytokines log_ADPN and log_IL‐10, but not with the proinflammatory cytokines log_IL‐6 and log_TNF‐α (Table 5, Figure 2). It also showed a significant correlation with STAI_1, but not with PA or EQ_VAS score. PA and STAI_1 showed a positive and negative significant correlation with EQ_VAS score, respectively, which was consistent with the multivariable regression results. However, contrary to the expected relationship between cytokines and mental health measures, PA did not show a positive significant correlation with anti‐inflammatory cytokines log_ADPN or log_IL‐10. However, STAI_1 score showed a significant correlation with log_IL‐6 and log_IL‐10, but not with log_ADPN or log_TNF‐α.

**TABLE 5:** Univariate correlations between clinical and cytokine variables.

	Physical	PA	NA	BIS	BAS	STAI_1	STAI_2	EQ_VAS	log_ADPN	log_CRP	log_IL‐6	log_lL‐10	log_IL‐1β	log_LEP	log_TNF‐α
Physical	1														
PA	0.123	1													
NA	0.191*	0.086	1												
BIS	0.117	0.111	0.356***	1											
BAS	0.210**	0.291***	0.149	0.395***	1										
STAI_1	0.177*	−0.173*	0.561***	0.196**	−0.089	1									
STAI_2	0.162*	0.037	0.503***	0.507***	0.279***	0.570***	1								
EQ_VAS	−0.114	0.261**	−0.260**	−0.089	0.160*	−0.409***	−0.271***	1							
log_ADPN	0.278***	0.023	0.060	0.147	0.187*	0.127	0.059	−0.056	1						
log_CRP	0.154*	0.125	0.054	0.041	0.149	−0.125	−0.092	−0.025	0.215**	1					
log_IL‐6	0.064	−0.199**	0.031	−0.151*	−0.140	0.194*	−0.105	−0.198**	0.354***	−0.068	1				
log_IL‐10	0.375***	0.008	0.033	0.071	0.203**	0.180*	0.121	−0.072	0.581***	0.234**	0.350***	1			
log_IL‐1β	0.149	−0.084	0.040	−0.010	0.114	0.118	0.013	−0.036	0.419***	0.207**	0.400***	0.524***	1		
log_LEP	−0.156*	0.095	−0.042	−0.074	−0.101	−0.168*	−0.086	0.073	−0.204**	0.046	0.005	−0.164*	0.005	1	
log_TNF‐α	0.141	−0.182*	−0.006	−0.192*	−0.150*	0.026	−0.167*	−0.138	0.220**	0.179*	0.459***	0.267***	0.312***	0.140	1

Abbreviations: ADPN, adiponectin; BIS‐BAS, behavioral Inhibition and Activation Systems; CRP, C‐reactive protein; EQ_VAS, EuroQol‐5 dimensions 5‐level visual analog scale; IL, interleukin; LEP, leptin; NA, negative affect; PA, positive affect; physical, physical disease severity; STAI, State–Trait Anxiety Inventory; TNF‐α, tumor necrosis factor‐α.

* *p* < 0.05.

** *p* < 0.01.

*** *p* < 0.001.

### 5.5 Structural Equation Model for Associations Between Physical Disease Severity, Cytokines, Mental Health Indicators, and QOL

**FIGURE 3:**
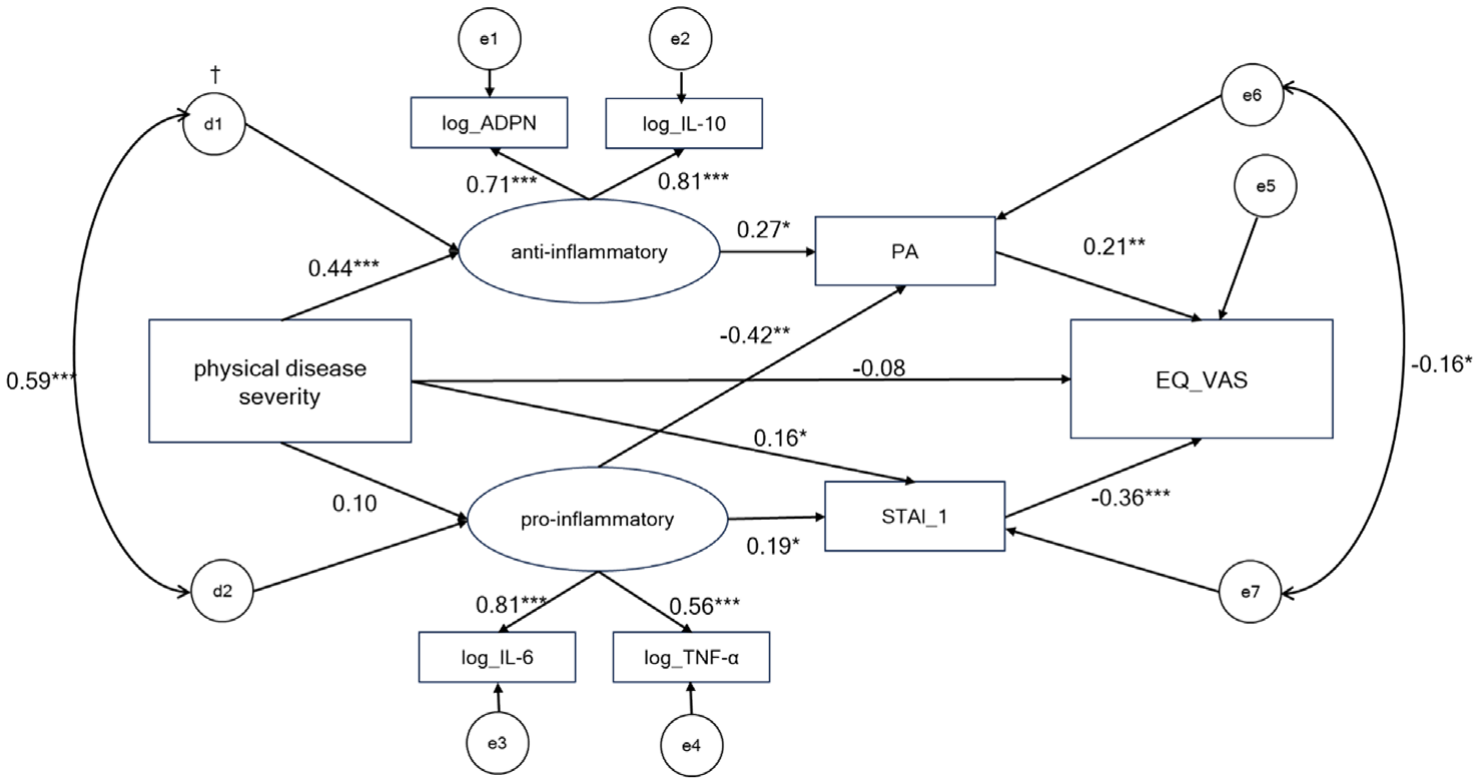
Hypothetical structural equation model. Hypothetical structural equation model for physical disease severity, cytokines (log_ADPN, log_IL‐10, log_IL‐6, log_TNF‐α), mental health indicators (PA, STAI) and QOL (EQ_VAS). In the path diagram, covariances are specified between the residuals of the latent variables representing anti‐inflammatory and proinflammatory markers, as well as between the residuals of PA and STAI_1. These covariances account for potential shared variance owing to commonality in residuals or overlapping constructs. The model fit was excellent. ^†^residuals. *p < 0.05, **p < 0.01, ***p < 0.001. *χ* ^2^ = 12.545, df = 13, *p* = 0.484; CFI = 1.000; RMSEA = 0.000. ADPN, adiponectin; EQ_VAS, EuroQol‐5 dimensions 5‐level visual analog scale; IL, interleukin; PA, positive affect; QOL, quality of life; STAI, state–trait anxiety inventory; TNF‐α, tumor necrosis factor‐α.

We constructed a model with three theoretically based paths: (i) physical disease severity → anti‐inflammatory cytokines → PA → QOL (EQ_VAS), (ii) physical disease severity → proinflammatory cytokines → situational anxiety (STAI_1) → QOL (EQ_VAS), and (iii) physical disease severity → QOL (EQ_VAS). We added two paths: (i) physical disease severity → situational anxiety (STAI_1), and (ii) proinflammatory cytokines → PA, taking into account the results of the intercorrelation analysis (Figure 3). The coefficient of the direct path from physical disease severity to QOL (EQ_VAS) was extremely low (−0.08), as was the path from physical disease severity to proinflammatory cytokines (0.10). All other paths were statistically significant. The model fit was excellent (*χ* ^2^ = 12.545, df = 13, *p* = 0.484; CFI = 1.000; RMSEA = 0.000).

As a large proportion of the participants were CHF patients, we compared the model structure and coefficients of each path in the model between the whole group of participants and CHF patients to determine if the inclusion of patients with other diseases affected the model structure or the coefficients of each path. When the structural model, which comprised 11 validated paths, was analyzed, the *p*‐value for the difference between the two groups was 0.995, indicating that the SEM was not significantly different. After analyzing each of the 11 paths, the between‐group differences were all nonsignificant (*p* = 0.285–0.999), indicating that the differences between the groups were small (Table 6).

**TABLE 6:** Path coefficients and group differences (all *n* = 173, CHF *n* = 146).

Path	Groups	Path coefficients	Standard error	*p*	CMIN	*p*
Physical disease severity → anti‐inflammatory	All	0.015	0.003	< 0.001	0.000	0.984
	CHF	0.015	0.003	< 0.001		
Physical disease severity → proinflammatory	All	0.034	0.030	0.250	0.030	0.862
	CHF	0.026	0.032	0.411		
PA → EQ_VAS	All	0.613	0.204	0.003	0.000	0.999
	CHF	0.613	0.234	0.009		
STAI → EQ_VAS	All	−0.789	0.154	< 0.001	0.308	0.579
	CHF	−0.916	0.168	< 0.001		
Physical disease severity → STAI	All	1.443	0.649	0.026	0.143	0.705
	CHF	1.799	0.679	0.008		
Physical disease severity → EQ_VAS	All	−1.458	1.334	0.274	0.025	0.874
	CHF	−1.770	1.430	0.216		
Proinflammatory → log_TNF‐α	All	0.437	0.093	< 0.001	1.144	0.285
	CHF	0.262	0.107	0.015		
Anti‐inflammatory → log_IL‐10	All	2.961	0.435	< 0.001	0.027	0.869
	CHF	3.072	0.519	< 0.001		
Anti‐inflammatory → PA	All	51.762	23.508	0.028	0.007	0.935
	CHF	49.006	21.440	0.022		
Proinflammatory → STAI	All	5.066	2.452	0.039	0.087	0.768
	CHF	4.104	2.433	0.092		
Proinflammatory → PA	All	−8.399	2.947	0.004	1.063	0.303
	CHF	−3.760	2.263	0.097		

Abbreviations: CHF, chronic heart failure; CMIN, chi‐square minimum value; EQ_VAS, EuroQol‐5 dimensions 5‐level visual analog scale; IL, interleukin; PA, positive affect; STAI, State–Trait Anxiety Inventory; TNF‐α, tumor necrosis factor‐α.

## 6 Discussion

Our main findings were that mental indicators are more strongly associated with subjective health‐related QOL than physical disease severity is, and that inflammatory cytokines are associated with mental health indicators, which is consistent with previous studies [11, 28, 29, 30, 31, 32, 33, 34]. In addition, we found that anti‐inflammatory cytokines increase in parallel with physical disease severity, and that this may lead to an improvement in mental resilience.

### 6.1 Participant Characteristics

Most of the NCD patients in the present study were CHF patients. The number of dementia patients in those with other NCDs was relatively high, so the age of patients in the other NCD group was higher than in the CHF group, and there were more patients who were not working or studying. There was no difference in physical disease severity between the groups, and subjective health‐related QOL was similar. In terms of mental health, PA was lower in the CHF group, but there was no difference in the other measures. Interestingly, with the exception of LEP, all inflammatory cytokine measures were higher in the CHF group, suggesting greater inflammatory activity in this group. The number of patients with comorbid mental disorders was unexpectedly low (two in the CHF group and one in the other group) compared with approximately 20% reported in a previous study of CHF patients [35]. This may reflect the fact that the presence of mental disorders is often overlooked, probably because of the overlap in symptoms common to CHF and depression and anxiety disorders [36]. Accurate diagnosis of these disorders is essential, as underdiagnosis has serious consequences.

### 6.2 Physical Disease Severity and Subjective Health‐Related QOL

In this study, we investigated the relationship between physical disease severity and subjective health‐related QOL, particularly the mediating effect of mental health indicators and inflammatory cytokines on this relationship. The multivariable regression analysis indicated a significant trend toward a negative correlation between physical disease severity and subjective health‐related QOL. This suggests that higher physical disease severity is associated with lower subjective health‐related QOL. Although the association between physical disease severity and EQ‐VAS showed only a significant trend, we tested an indirect pathway based on theoretical and empirical grounds. We created a hypothetical model that included inflammatory cytokines (TNF‐α, IL‐6, IL‐10, ADPN) and mental health indicators (PA, STAI_1 scores) as mediating factors. The direct path coefficient from physical disease severity to subjective health‐related QOL was extremely low (−0.08), suggesting a mediating effect of these factors.

Our findings suggest that physical disease severity is associated with lower QOL via worsening mental health. In contrast to objective QOL measures, the QOL indicator used in this study was the EQ_VAS, which is a subjective measure of health expressed on a VAS and is more likely to reflect psychological distress than objective measures of the severity of movement and activity limitations due to physical diseases. In a previous study of congestive heart failure, QOL measured by the 36‐item Short Form Health Survey (SF‐36) significantly decreased with NYHA functional class, but the variance in the SF‐36 scale scores explained by NYHA functional class was small (10%–28%), except for physical functioning (40%) [37]. Another study reported that limitations in the QOL and psychological well‐being of patients with dilated cardiomyopathy were only partly explained by the symptoms and severity of underlying disease [38]. In a study of dementia with Lewy bodies, when the physical and mental components of the SF‐8 (used to measure QOL) were examined separately for contributing factors, motor symptoms had a significant effect on the former and depressive symptoms only on the latter [39]. Considering these findings, objective physical symptoms may affect satisfaction with physical functioning but not satisfaction with other life aspects, suggesting the importance of measuring subjective general health‐related QOL (which may not reflect physical disease severity) in addition to using objective measures [32]. A stronger association between mental health indicators (compared with physical disease severity) and QOL (particularly the mental aspect of QOL) has also been found in patients with other NCDs such as asthma and diabetes [33, 34]. In addition, noncognitive symptoms such as functional impairment and depressive symptoms seem more relevant to QOL than cognitive symptoms in patients with AD and Lewy body dementia, perhaps because of the effect of noncognitive symptoms on subjective QOL and disease burden in patients with dementia [40].

### 6.3 Physical Disease Severity and Mental Health Indicators

Regarding the relationship between physical disease severity and mental health indicators, physical disease severity showed a direct relationship with the negative mental health variable of situational anxiety (STAI_1 score), and proinflammatory cytokines, represented by IL‐6 and TNF‐α levels, did not mediate between these factors. However, proinflammatory cytokines had significant negative and positive effects on PA and STAI_1, respectively, independent of physical disease severity.

Many previous study findings have suggested that mental disorders are likely to occur in conjunction with NCDs, and that the severity of physical disease is related to mental health symptoms [6, 28, 36, 41, 42, 43, 44]. However, the mediators of the relationship between the physical and mental states of NCDs are not necessarily clear. In diabetes, for example, it is known that depression can easily cause a decline in glucose tolerance owing to overactivity of the hypothalamus–pituitary–adrenocortical axis and the sympathetic nervous system [7, 28], and that psychological distress and self‐management burden caused by diabetes are strongly associated with depression [29]. It is therefore likely that psychological distress may lead to mental disorders such as depression, not only in diabetes but also in other NCDs. However, it is possible that various cytokines associated with the inflammatory response that commonly occurs in NCDs may affect mental health. In previous studies of CHF and diabetes patients, depressive symptoms were associated with higher levels of the proinflammatory cytokines TNF‐α, IL‐6, and CRP, and lower levels of the anti‐inflammatory cytokines IL‐10 and ADPN [45, 46, 47, 48, 49, 50].

### 6.4 Inflammatory Cytokines as Mediators Between Physical Disease and Mental Health

Previous studies have demonstrated activation of the inflammatory system resulting in the production and release of systemic inflammatory cytokines in patients with heart failure [10, 51, 52], diabetes [30, 53], and even neurodegenerative diseases that are normally protected by the blood–brain barrier, including AD and PD [31, 54, 55]. The disruption of neurovascular homeostasis, namely, the increased permeability of the blood–brain barrier and the infiltration of systemic inflammatory mediators into the brain, is a pathological factor in neurodegenerative diseases [55]. Although previous studies of the inflammatory process in the central nervous system have focused on microglial production of proinflammatory cytokines, a large body of evidence indicates that microglia also produce anti‐inflammatory cytokines such as TGF‐β, IL‐10, and IL‐1 receptor antagonist [56]. Anti‐inflammatory cytokines such as TGF‐β and IL‐10 inhibit microglial activation through their ability to inhibit antigen presentation and proinflammatory cytokines, chemokines, and reactive oxygen intermediates [57, 58]. In heart failure, the production of proinflammatory cytokines and chemokines increases in the early stages of tissue damage, and after the rapid influx of neutrophils and monocytes into the damaged area, the influx of regulatory T cells (Tregs) and B cells and the production of anti‐inflammatory cytokines such as lipoxins, TGF‐β, and IL‐10 lead to the repair phase [52]. Myocardial inflammation is persistent in the chronic phase, as proinflammatory cytokines and chemokines have been detected in the hearts of CHF patients [59]. Similarly, in a systemic disease such as T1DM, the proinflammatory cytokine TNF‐α and the anti‐inflammatory cytokine IL‐10 are elevated in patients with poor glycemic control [60]. A review of the relationship between T1DM and major depressive disorder suggested that patients with T1DM have increased levels of cytokines IL‐4, IL‐6, IL‐10, and TNF‐α as a result of beta cell destruction and hyperglycemia, and that increased plasma IL‐6, IL‐1, and TNF‐α induce symptoms associated with major depressive disorder [28]. A meta‐analytic study suggested that elevated levels of IL‐6 were associated with an increased incidence of T1DM, whereas elevated levels of IL‐10 were observed in people with newly diagnosed T1DM [30], possibly owing to the protective response of the immune system to the onset of the disease.

In the present study, the proinflammatory cytokines IL‐6 and TNF‐α affected both emotions and anxiety, but unlike the anti‐inflammatory cytokines IL‐10 and ADPN, they were not related to physical disease severity. As noted above, the chronic phase of heart failure is associated with persistent proinflammatory activity, which is thought to contribute to adverse clinical outcomes [52]. Several studies have identified a significant relationship between physical disease severity and levels of proinflammatory cytokines [28, 30, 59, 60], but not all studies have found this relationship [10]. Persistent proinflammatory activity in CHF may be provoked by the ongoing myocardial injury induced by various stimuli, such as activation of the sympathetic nervous system and the renin–angiotensin–aldosterone system, autoantibodies, heat shock protein, microbial antigen, bacterial lipopolysaccharide, shear and oxidative stress, and hypoxia, which may have a subthreshold effect on current physical status [52, 61]. However, it is noteworthy that in the present study, anti‐inflammatory activity was related to physical disease severity and may play a protective role against the damage caused by worsening physical status [51]. This finding is consistent with previous studies showing that patients with higher NYHA functional class have significantly higher blood concentrations of ADPN, TNF‐β, and IL‐10 [53, 62]. Active secretion of the anti‐inflammatory cytokines ADPN, TGF‐β, and IL‐10 by Tregs in severe physical states suppresses the immune response of proinflammatory cytokines [63, 64] and may contribute to the unclear relationship between proinflammatory cytokine blood levels and physical disease severity.

Given the positive effects of anti‐inflammatory cytokines on emotions and their potential to suppress excessive inflammatory responses and reduce exacerbations of NCDs, increasing the action of Tregs responsible for the secretion of such cytokines may contribute to the treatment of psychiatric symptoms complicating NCDs.

Limitations of this study include the fact that most participants were CHF patients, a population that is not representative of the NCD population as a whole, and that the measures used to assess physical disease severity differed by disease. It should be noted that the assessment of CHF, PD, and SCD focuses on activities of daily living, whereas T1DM assessment uses biomarkers, and AD and MCI assessments use cognitive impairment tests. Therefore, the implications of disease severity may differ accordingly. However, the SEM structure did not change significantly when restricted to CHF patients, and the results appear to be relatively robust. Nevertheless, future between‐group comparisons should include higher numbers of non‐CHF patients. Finally, we could not control for the effects of comorbid autoimmune or inflammatory diseases, nor could we control for medications such as steroids that may influence the inflammatory system. Since NCDs are believed to be associated with chronic inflammation, and patients are often treated with such medications, controlling for the effects of comorbidities and medications is difficult.

## 7 Conclusions

We investigated the factors that mediate the association between the severity of NCDs and health‐related QOL. The results showed that mental health indicators such as anxiety and positive emotions mediate both variables, and that proinflammatory cytokines such as IL‐6 and TNF‐α may reduce subjective health‐related QOL by increasing anxiety and suppressing positive emotions, but are not particularly related to the severity of physical disease. In addition, anti‐inflammatory cytokines such as IL‐10 and ADPN are activated as the severity of physical disease increases, and are likely to protect against physical disease and enhance positive emotions, potentially increasing health‐related QOL and resilience.

## Author Contributions

Conception and design of the study: K.N., M.A., F.Y., R.S., H.K., and M.F.; acquisition and analysis of data: M.A., C.I., T.N., T.T., F.Y., R.S., H.K., M.F., and K.H.; interpretation of data: K.N., Y.T., Y.U., Y.O., and Y.M.; drafting the manuscript or figures: K.N., R.K., and M.O.; reviewing the work critically: M.A., C.I., T.N., T.T., F.Y., R.S., H.K., M.F., K.H., Y.T., Y.U., Y.O., Y.M., R.K., and M.O.; final approval: K.N., M.A., C.I., T.N., T.T., F.Y., R.S., H.K., M.F., K.H., Y.T., Y.U., Y.O., Y.M., R.K., and M.O.; agreement to be accountable: K.N., M.A., C.I., T.N., T.T., F.Y., R.S., H.K., M.F., K.H., Y.T., Y.U., Y.O., Y.M., R.K., and M.O.

## Ethics Statement

The protocol for this research project has been approved by the Ethics Committee of the National Center of Neurology and Psychiatry (Approval No. B2020‐110) and it conforms to the provisions of the Declaration of Helsinki.

## Conflicts of Interest

K.N. received grants paid to his institution from Shionogi & Co. Ltd., Sumitomo Pharma Co. Ltd., Otsuka Pharmaceutical Co., Meiji‐Seika Pharma Co. Ltd., Janssen Pharmaceutical, Mitsubishi Tanabe Pharma Corp., Nippon Boehringer Ingelheim Co. Ltd., and Mochida Pharmaceutical Co. Ltd. in the past 36 months; payment/honoraria from Sumitomo Pharma Co. Ltd., Otsuka Pharmaceutical Co., Meiji‐Seika Pharma Co. Ltd., Janssen Pharmaceutical, Mitsubishi Tanabe Pharma Corp., Takeda Pharmaceutical Co. Ltd., Lundbeck Japan, Viatris, Eisai Co. Ltd., Nippon Boehringer Ingelheim Co. Ltd., and Mochida Pharmaceutical Co. Ltd. in the past 36 months; and support for attending meetings and/or travel from Sumitomo Pharma Co. Ltd., Otsuka Pharmaceutical Co., Meiji‐Seika Pharma Co. Ltd., Janssen Pharmaceutical, Mitsubishi Tanabe Pharma Corp., Takeda Pharmaceutical Co. Ltd., Nippon Boehringer Ingelheim Co. Ltd., and Mochida Pharmaceutical Co. Ltd. in the past 36 months. Y.M. owns Mitsubishi Chemical Group Corporation stock. Y.T. received grants paid to his institution from Nihon Medi‐Physics Co. Ltd., Takeda Pharmaceutical Co. Ltd., Astellas Co. Ltd., Eisai Co. Ltd., Asahi Kasei Pharma Co. Ltd., Ono Pharma Co. Ltd., Daiichi Sankyo Co. Ltd., Mitsubishi Tanabe Pharma Co. Ltd., and Nippon Shinyaku Co. Ltd. in the last 36 months; payment/honoraria from Daiichi Sankyo Co. Ltd., Takeda Pharmaceutical Co. Ltd., Sumitomo Pharma Co. Ltd., Otsuka Pharmaceutical Co., Nihon Medi‐Physics Co. Ltd., Kyowa Kirin Co. Ltd., Fujimoto Pharmaceutical Co., Abbvie Inc., Amgen Inc., Ono Pharma Co. Ltd., and Alnylam Pharmaceutical Inc. in the last 36 months. Dr. Kazuyuki Nakagome is an Editorial Board member of Neuropsychopharmacology Reports and a coauthor of this article. To minimize bias, he was excluded from all editorial decision‐making related to the acceptance of this article for publication.

## Consent

The patients who participated in this study gave informed consent, and patient anonymity was preserved.

## Data Availability

The data that support the findings of this study are available on request from the corresponding author. The data are not publicly available due to privacy or ethical restrictions. The research plan approved by the Ethics Committee clearly states that secondary use of individual data requires obtaining renewed consent depending on the recipient and purpose of use, making it difficult to register individual data without specifying the purpose.

## References

[1] World Health Organizations , “World Health Statistics 2023: Monitoring Health for the SDGs, Sustainable Development Goals. 2023,” (2023), cited December 6, 2024, https://www.psychiatrist.com/jcp/schizophrenia/patient‐centered‐assessment‐in‐schizophrenia/.

[2] J. Fisher , G. Fones , Y. Arivalagan , I. Ahmadpour , S. Akselrod , and M. Olsen , “WHO Framework on Meaningful Engagement: A Transformational Approach to Integrate Lived Experience in the Noncommunicable Disease and Mental Health Agenda,” PLOS Global Public Health 4 (2024): e0002312. 38809940 10.1371/journal.pgph.0002312 PMC11135697

[3] R. M. Carney and K. E. Freedland , “New Perspectives on Treatment of Depression in Coronary Heart Disease,” Psychosomatic Medicine 85 (2023): 474–478. 37234020 10.1097/PSY.0000000000001219 PMC10524988

[4] N. Frasure‐Smith , F. Lespérance , and M. Talajic , “Depression Following Myocardial Infarction. Impact on 6‐Month Survival,” JAMA 270 (1993): 1819–1825. 8411525

[5] A. Meijer , H. J. Conradi , E. H. Bos , B. D. Thombs , J. P. van Melle , and P. de Jonge , “Prognostic Association of Depression Following Myocardial Infarction With Mortality and Cardiovascular Events: A Meta‐Analysis of 25 Years of Research,” General Hospital Psychiatry 33 (2011): 203–216. 21601716 10.1016/j.genhosppsych.2011.02.007

[6] M. Park and C. F. Reynolds , “Depression Among Older Adults With Diabetes Mellitus,” Clinics in Geriatric Medicine 31 (2015): 117–137. 25453305 10.1016/j.cger.2014.08.022 PMC4254540

[7] N. Schmitz , S. S. Deschênes , R. J. Burns , et al., “Depression and Risk of Type 2 Diabetes: The Potential Role of Metabolic Factors,” Molecular Psychiatry 21 (2016): 1726–1732. 26903269 10.1038/mp.2016.7

[8] J. da Silva , M. Gonçalves‐Pereira , M. Xavier , and E. B. Mukaetova‐Ladinska , “Affective Disorders and Risk of Developing Dementia: Systematic Review,” British Journal of Psychiatry 202 (2013): 177–186. 10.1192/bjp.bp.111.101931 23457181

[9] B. W. J. H. Penninx , F. Lamers , R. Jansen , et al., “Immuno‐Metabolic Depression: From Concept to Implementation,” Lancet Regional Health Europe 48 (2025): 48. 10.1016/j.lanepe.2024.101166 PMC11721223 39801616

[10] G. Torre‐Amione , “Immune Activation in Chronic Heart Failure,” American Journal of Cardiology 95 (2005): 3C–8C. 10.1016/j.amjcard.2005.03.006 15925558

[11] G. L. Xiong , K. Prybol , S. H. Boyle , et al., “Inflammation Markers and Major Depressive Disorder in Patients With Chronic Heart Failure: Results From the Sertraline Against Depression and Heart Disease in Chronic Heart Failure Study,” Psychosomatic Medicine 77 (2015): 808–815. 26186432 10.1097/PSY.0000000000000216 PMC4565768

[12] C. Kraus , B. Kadriu , R. Lanzenberger , C. A. Zarate , and S. Kasper , “Prognosis and Improved Outcomes in Major Depression: A Review,” Translational Psychiatry 9 (2019): 127. 30944309 10.1038/s41398-019-0460-3 PMC6447556

[13] The Criteria Committee of the New York Heart Association , Nomenclature and Criteria for Diagnosis of Diseases of the Heart and Great Vessels, 9th ed. (Little, Brown & Co, 1994).

[14] Y. Shinohara , K. Minematsu , T. Amano , and Y. Ohashi , “Reliability of Modified Rankin Scale‐Introduction of a Guidance Scheme and a Questionnaire Written in Japanese,” Nosotchu 29 (2007): 6–13.

[15] M. Sugishita , Mini Mental State Examination‐Japanese (MMSE‐J) (Nihon Bunka Kagakusha Co., Ltd, 2012).

[16] E. Araki , A. Goto , T. Kondo , et al., “Japanese Clinical Practice Guideline for Diabetes 2019,” Diabetology International 11 (2020): 165–223. 32802702 10.1007/s13340-020-00439-5 PMC7387396

[17] D. Watson , L. A. Clark , and A. Tellegen , “Development and Validation of Brief Measures of Positive and Negative Affect: The PANAS Scales,” Journal of Personality and Social Psychology 54 (1988): 1063–1070. 3397865 10.1037//0022-3514.54.6.1063

[18] C. S. Carver and T. L. White , “Behavioral Inhibition, Behavioral Activation, and Affective Responses to Impending Reward and Punishment: The BIS/BAS Scales,” Journal of Personality and Social Psychology 67 (1994): 319–333.

[19] Y. Takahashi , S. Yamagata , N. Kijima , K. Shigematsu , Y. Ono , and J. Ando , “Gray's Temperament Model: Development of Japanese Version of BIS/BAS Scales and a Behavior Genetic Investigation Using the Twin Method,” Japanese Journal of Personality 15 (2007): 276–289.

[20] C. Spielberger , R. Gorsuch , and R. Lushene , Manual for the State‐Trait Anxiety Inventory (Self‐Evaluation Questionnaire) (Consulting Psychologists Press, 1970).

[21] H. Shimizu and K. Imae , “Development of the Japanese Version of State‐Trait Anxiety Inventory,” Japanese Journal of Educational Psychology 29 (1981): 348–353.

[22] M. Herdman , C. Gudex , A. Lloyd , et al., “Development and Preliminary Testing of the New Five‐Level Version of EQ‐5D (EQ‐5D‐5L),” Quality of Life Research 20 (2011): 1727–1736. 21479777 10.1007/s11136-011-9903-x PMC3220807

[23] S. Ikeda , T. Shiroiwa , A. Igarashi , et al., “Developing a Japanese Version of the EQ‐5D‐5L Value Set,” Journal of the National Institute of Public Health 64 (2015): 47–55.

[24] A. Sato and A. Yasuda , “Development of the Japanese Version of Positive and Negative Affect Schedule (PANAS) Scales,” Japanese Journal of Personality 9 (2001): 138–139.

[25] P. Bentler , “Comparative Fit Indexes in Structural Models,” Psychological Bulletin 107 (1990): 238–246. 2320703 10.1037/0033-2909.107.2.238

[26] L.‐t. Hu and P. Bentler , “Cutoff Criteria for Fit Indexes in Covariance Structure Analysis: Conventional Criteria Versus New Alternatives,” Structural Equation Modeling 6 (1999): 1–55.

[27] M. Haluzík , J. Pařízková , and M. M. Haluzík , “Adiponectin and Its Role in the Obesity‐Induced Insulin Resistance and Related Complications,” Physiological Research 53 (2004): 123–129. 15046547

[28] D. J. Korczak , S. Pereira , K. Koulajian , A. Matejcek , and A. Giacca , “Type 1 Diabetes Mellitus and Major Depressive Disorder: Evidence for a Biological Link,” Diabetologia 54 (2011): 2483–2493. 21789690 10.1007/s00125-011-2240-3

[29] K. K. Hood , J. M. Lawrence , A. Anderson , et al., “Metabolic and Inflammatory Links to Depression in Youth With Diabetes,” Diabetes Care 35 (2012): 2443–2446. 23033243 10.2337/dc11-2329 PMC3507554

[30] Z. Jin , Q. Zhang , K. Liu , et al., “The Association Between Interleukin Family and Diabetes Mellitus and Its Complications: An Overview of Systematic Reviews and Meta‐Analyses,” Diabetes Research and Clinical Practice 210 (2024): 111615. 38513987 10.1016/j.diabres.2024.111615

[31] A. Rauf , H. Badoni , T. Abu‐Izneid , et al., “Neuroinflammatory Markers: Key Indicators in the Pathology of Neurodegenerative Diseases,” Molecules 27 (2022): 3194. 35630670 10.3390/molecules27103194 PMC9146652

[32] G. Majani , A. Pierobon , A. Giardini , et al., “Relationship Between Psychological Profile and Cardiological Variables in Chronic Heart Failure,” European Heart Journal 20 (1999): 1579–1586. 10529326 10.1053/euhj.1999.1712

[33] K. L. Lavoie , A. Cartier , M. Labrecque , et al., “Are Psychiatric Disorders Associated With Worse Asthma Control and Quality of Life in Asthma Patients?,” Respiratory Medicine 99 (2005): 1249–1257. 16140225 10.1016/j.rmed.2005.03.003

[34] N. Panahi , M. Ahmadi , M. Hosseinpour , et al., “The Association Between Quality of Life and Diabetes: The Bushehr Elderly Health Program,” BMC Geriatrics 24 (2024): 267. 38500039 10.1186/s12877-024-04878-6 PMC10949763

[35] T. Rutledge , V. Reis , S. Linke , B. Greenberg , and P. J. Mills , “Depression in Heart Failure a Meta‐Analytic Review of Prevalence, Intervention Effects, and Associations With Clinical Outcomes,” Journal of the American College of Cardiology 48 (2006): 1527–1537. 17045884 10.1016/j.jacc.2006.06.055

[36] C. M. Celano , A. C. Villegas , A. M. Albanese , H. K. Gaggin , and J. C. Huffman , “Depression and Anxiety in Heart Failure: A Review,” Harvard Review of Psychiatry 26 (2018): 175–184. 29975336 10.1097/HRP.0000000000000162 PMC6042975

[37] J. Juenger , D. Schellberg , S. Kraemer , A. Haunstetter , C. Zugck , and W. Herzog , “Health Related Quality of Life in Patients With Congestive Heart Failure: Comparison With Other Chronic Diseases and Relation to Functional Variables,” Heart 87 (2002): 235–241. 11847161 10.1136/heart.87.3.235 PMC1767036

[38] A. Steptoe , A. Mohabir , N. G. Mahon , and W. J. Mckenna , “Health Related Quality of Life and Psychological Wellbeing in Patients With Dilated Cardiomyopathy,” Heart 83 (2000): 645–650. 10814621 10.1136/heart.83.6.645 PMC1760850

[39] S. Toya , M. Hashimoto , Y. Manabe , H. Yamakage , and M. Ikeda , “Factors Associated With Quality of Life in Patients With Dementia With Lewy Bodies: Additional Analysis of a Cross‐Sectional Study,” Journal of Alzheimer's Disease 100 (2024): 525–538. 10.3233/JAD-231302 PMC11307033 38875033

[40] M. van de Beek , I. van Steenoven , I. H. G. B. Ramakers , et al., “Trajectories and Determinants of Quality of Life in Dementia With Lewy Bodies and Alzheimer's Disease,” Journal of Alzheimer's Disease 70 (2019): 387–395. 10.3233/JAD-190041 PMC6839497 31177218

[41] H. Carreira , R. Williams , H. Dempsey , S. Stanway , L. Smeeth , and K. Bhaskaran , “Quality of Life and Mental Health in Breast Cancer Survivors Compared With Non‐Cancer Controls: A Study of Patient‐Reported Outcomes in the United Kingdom,” Journal of Cancer Survivorship 15 (2021): 564–575. 33089480 10.1007/s11764-020-00950-3 PMC8272697

[42] N. Schmitz , S. Deschênes , R. Burns , and K. J. Smith , “Depressive Symptoms and Glycated Hemoglobin A1c: A Reciprocal Relationship in a Prospective Cohort Study,” Psychological Medicine 46 (2016): 945–955. 26620309 10.1017/S0033291715002445

[43] N. Tsabedze , J. L. H. Kinsey , D. Mpanya , V. Mogashoa , E. Klug , and P. Manga , “The Prevalence of Depression, Stress and Anxiety Symptoms in Patients With Chronic Heart Failure,” International Journal of Mental Health Systems 15 (2021): 44. 33980322 10.1186/s13033-021-00467-x PMC8114712

[44] H. Korkmaz , S. Korkmaz , and M. Çakar , “Suicide Risk in Chronic Heart Failure Patients and Its Association With Depression, Hopelessness and Self Esteem,” Journal of Clinical Neuroscience 68 (2019): 51–54. 31375305 10.1016/j.jocn.2019.07.062

[45] J. T. Parissis , S. Adamopoulos , A. Rigas , et al., “Comparison of Circulating Proinflammatory Cytokines and Soluble Apoptosis Mediators in Patients With Versus Without Symptoms of Depression,” American Journal of Cardiology 94 (2004): 1326–1328. 15541260 10.1016/j.amjcard.2004.07.127

[46] J. T. Parissis , D. Farmakis , M. Nikolaou , et al., “Plasma B‐Type Natriuretic Peptide and Anti‐Inflammatory Cytokine Interleukin‐10 Levels Predict Adverse Clinical Outcome in Chronic Heart Failure Patients With Depressive Symptoms: A 1‐Year Follow‐Up Study,” European Journal of Heart Failure 11 (2009): 967–972. 19789400 10.1093/eurjhf/hfp125

[47] J. P. Empana , D. H. Sykes , G. Luc , et al., “Contributions of Depressive Mood and Circulating Inflammatory Markers to Coronary Heart Disease in Healthy European Men: The Prospective Epidemiological Study of Myocardial Infarction (PRIME),” Circulation 111 (2005): 2299–2305. 15867179 10.1161/01.CIR.0000164203.54111.AE

[48] K. W. Davidson , J. E. Schwartz , S. A. Kirkland , et al., “Relation of Inflammation to Depression and Incident Coronary Heart Disease (From the Canadian Nova Scotia Health Survey [NSHS95] Prospective Population Study),” American Journal of Cardiology 103 (2009): 755–761. 19268727 10.1016/j.amjcard.2008.11.035 PMC2905847

[49] C. Herder , M. Carstensen , and D. M. Ouwens , “Anti‐Inflammatory Cytokines and Risk of Type 2 Diabetes,” Diabetes, Obesity and Metabolism 15 (2013): 39–50. 10.1111/dom.12155 24003920

[50] C. Herder , A. Schmitt , F. Budden , et al., “Association Between Pro‐ and Anti‐Inflammatory Cytokines and Depressive Symptoms in Patients With Diabetes‐Potential Differences by Diabetes Type and Depression Scores,” Translational Psychiatry 7 (2017): 1. 10.1038/s41398-017-0009-2 PMC5843637 29520075

[51] M. Bartekova , J. Radosinska , M. Jelemensky , and N. S. Dhalla , “Role of Cytokines and Inflammation in Heart Function During Health and Disease,” Heart Failure Reviews 23 (2018): 733–758. 29862462 10.1007/s10741-018-9716-x

[52] L. Adamo , C. Rocha‐Resende , S. D. Prabhu , and D. L. Mann , “Reappraising the Role of Inflammation in Heart Failure,” Nature Reviews. Cardiology 17 (2020): 269–285. 31969688 10.1038/s41569-019-0315-x

[53] J. George , S. Patal , D. Wexler , et al., “Circulating Adiponectin Concentrations in Patients With Congestive Heart Failure,” Heart 92 (2006): 1420–1424. 16621874 10.1136/hrt.2005.083345 PMC1861042

[54] H. B. Stolp and K. M. Dziegielewska , “Review: Role of Developmental Inflammation and Blood‐Brain Barrier Dysfunction in Neurodevelopmental and Neurodegenerative Diseases,” Neuropathology and Applied Neurobiology 35 (2009): 132–146. 19077110 10.1111/j.1365-2990.2008.01005.x

[55] L. M. Collins , A. Toulouse , T. J. Connor , and Y. M. Nolan , “Contributions of Central and Systemic Inflammation to the Pathophysiology of Parkinson's Disease,” Neuropharmacology 62 (2012): 2154–2168. 22361232 10.1016/j.neuropharm.2012.01.028

[56] Y. S. Kim and T. H. Joh , “Microglia, Major Player in the Brain Inflammation: Their Roles in the Pathogenesis of Parkinson's Disease,” Experimental & Molecular Medicine 38 (2006): 333–347. 16953112 10.1038/emm.2006.40

[57] K. Frei , H. Lins , C. Schwerdel , and A. Fontana , “Antigen Presentation in the Central Nervous System. The Inhibitory Effect of IL‐10 on MHC Class II Expression and Production of Cytokines Depends on the Inducing Signals and the Type of Cell Analyzed,” Journal of Immunology 152 (1994): 2720–2728. 8144879

[58] G. M. O'Keefe , V. T. Nguyen , and E. N. Benveniste , “Class II Transactivator and Class II MHC Gene Expression in Microglia: Modulation by the Cytokines TGF‐β, IL‐4, IL‐13 and IL‐10,” European Journal of Immunology 29 (1999): 1275–1285. 10229095 10.1002/(SICI)1521-4141(199904)29:04<1275::AID-IMMU1275>3.0.CO;2-T

[59] G. Torre‐Amione , S. Kapadia , J. Lee , et al., “Tumor Necrosis Factor‐Alpha and Tumor Necrosis Factor Receptors in the Failing Human Heart,” Circulation 93 (1996): 704–711. 8640999 10.1161/01.cir.93.4.704

[60] M. C. Foss‐Freitas , N. T. Foss , D. M. Rassi , E. A. Donadi , and M. C. Foss , “Evaluation of Cytokine Production From Peripheral Blood Mononuclear Cells of Type 1 Diabetic Patients: Importance of the Methodologic Approach,” Annals of the New York Academy of Sciences 1150 (2008): 290–296. 19120315 10.1196/annals.1447.053

[61] P. Aukrust , L. Gullestad , T. Ueland , J. K. Damås , and A. Yndestad , “Inflammatory and Anti‐Inflammatory Cytokines in Chronic Heart Failure: Potential Therapeutic Implications,” Annals of Medicine 37 (2005): 74–85. 16026115 10.1080/07853890510007232

[62] D. L. Dixon , K. M. Griggs , A. D. Bersten , and C. G. de Pasquale , “Systemic Inflammation and Cell Activation Reflects Morbidity in Chronic Heart Failure,” Cytokine 56 (2011): 593–599. 21924921 10.1016/j.cyto.2011.08.029

[63] S. M. Cabrera , M. R. Rigby , and R. G. Mirmira , “Targeting Regulatory T Cells in the Treatment of Type 1 Diabetes Mellitus,” Current Molecular Medicine 12 (2012): 1261–1272. 22709273 10.2174/156652412803833634 PMC3709459

[64] Y. Lu , N. Xia , and X. Cheng , “Regulatory T Cells in Chronic Heart Failure,” Frontiers in Immunology 12 (2021): 12. 10.3389/fimmu.2021.732794 PMC8493934 34630414

